# Compressive Postoperative Seromas Causing Delayed Neurological Deterioration Following Cervical Laminectomy and Instrumented Fusion

**DOI:** 10.7759/cureus.46326

**Published:** 2023-10-01

**Authors:** Kelvin Kah Ho Lor, Joshua Decruz, Mu Liang Ang, Boon Chuan Pang, Eugene Yang

**Affiliations:** 1 Orthopaedic Surgery, Khoo Teck Puat Hospital, Singapore, SGP; 2 Orthopaedic Surgery, Woodlands Health, Singapore, SGP; 3 Neurosurgery, Khoo Teck Puat Hospital, Singapore, SGP

**Keywords:** degenerative cervical myelopathy, neurological complication, posterior cervical decompression and fusion, bmp-2, post-operative seroma

## Abstract

Compressive postoperative seromas in the cervical spine are a rare but significant complication following cervical laminectomy and instrumented fusion. There is a paucity of cases reported in the literature, with a majority of the reported cases attributing seroma formation to the use of recombinant human bone morphogenetic protein-2 (rhBMP-2). In this article, we report four cases of compressive postoperative seroma in the absence of rhBMP-2 use and highlight similarities in their clinical presentations. We postulate that seroma formation is a significant complication of the dead space that results following posterior instrumentation in the cervical spine, with or without the use of rhBMP-2. The typical presentation is one of the gradual delayed neurological deterioration several days following the index surgery and after drain removal. Neurological deterioration can be reversed rapidly with early recognition and drainage of the seroma.

## Introduction

Postoperative seromas causing spinal cord compression in the cervical spine are a rare but significant complication following cervical laminectomy and instrumented fusion. While seromas are not uncommon following posterior spinal surgery, the accumulation of sufficient amounts of fluid to cause mass effect on the spinal cord and neurological deficits and/or myelopathy are much rarer. A large majority of information in the literature on this condition is from case reports that focused on the role of recombinant human bone morphogenetic protein-2 (rhBMP-2) in seroma formation [1-4], rhBMP-2 being an osteo-inductive agent with increasing use in spine surgery to achieve spinal fusion, although two subsequent case reports [5,6] also noted this complication in the absence of rhBMP-2. Overall, there is a paucity of information about this complication. The purpose of this article is to report on four cases of postoperative seroma formation causing cord compression and neurological deterioration in the absence of rhBMP-2 use. We highlight the typical clinical presentation encountered and discuss the likely pathophysiology and treatment options available.

## Case presentation

Case 1

**Figure 1:**
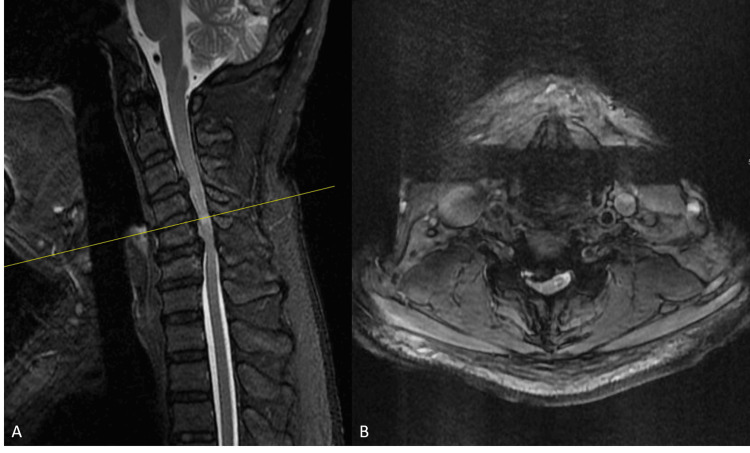
Preoperative sagittal (A) and axial (B) MRI showing multilevel cervical cord compression, C4-C5 myelomalacia, and mild kyphosis

A 67-year-old gentleman with a significant past medical history of severe cardiomyopathy on dual antiplatelet therapy, diabetes mellitus, and chronic kidney disease presented with a one-year history of predominantly left-sided upper and lower limb dysfunction requiring a wheelchair for ambulation. On examination, he had significant spasticity and weakness over his left upper and lower limbs (3-4/5), with positive Hoffman’s sign bilaterally. Magnetic resonance imaging (MRI) of the brain revealed no stroke disease to account for his predominantly unilateral symptoms, while MRI of the cervical spine demonstrated multilevel degenerative changes with severe cord compression over C3-C6 and myelomalacia at C4-C5 as well as a mild kyphotic deformity over the involved segments (Figure 1, Panels A and B).

He underwent C3-C6 laminectomy and instrumented fusion using lateral mass screws. Complete laminectomies of C3-C6 were performed. Fusion was performed by on-laying demineralized bone matrix (DBM) putty mixed with morselized autograft from the laminectomies in the lateral gutters following decortication. Hemostasis was secured using an oxidized regenerated cellulose (ORC) hemostatic agent, and vancomycin powder was applied to the implants. The wound was closed in layers over a closed suction drain placed deep into the fascia. Surgery was uneventful with stable neuromonitoring throughout.

**Figure 2:**
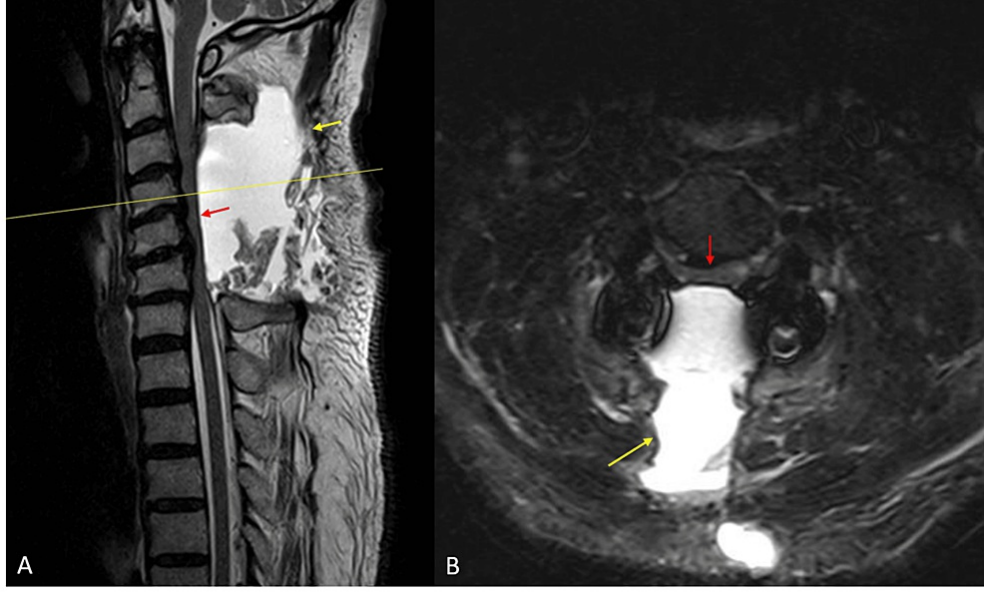
Sagittal (A) and axial (B) MRI on postoperative day eight demonstrating a large fluid collection (yellow arrows) compressing the spinal cord (red arrows)

The patient awoke from surgery with no new neurological deficits, and the drain was removed on postoperative day three after the drain output turned serous and drainage was 60 mL over 24 hrs. Initial rehabilitation in the first week progressed uneventfully. On postoperative day seven, the patient complained of mild weakness of his left-hand grip strength (4/5), which had previously been intact. This subsequently progressed to involve the previously uninvolved right upper limb with loss of motor power in elbow flexion (4/5) and extension (3/5). An MRI was performed in view of his progressive upper limb neurological deterioration, which revealed a large posterior fluid collection that was homogeneously T2 hyperintense compressing the spinal cord (Figure 2, Panels A and B).

**Figure 3:**
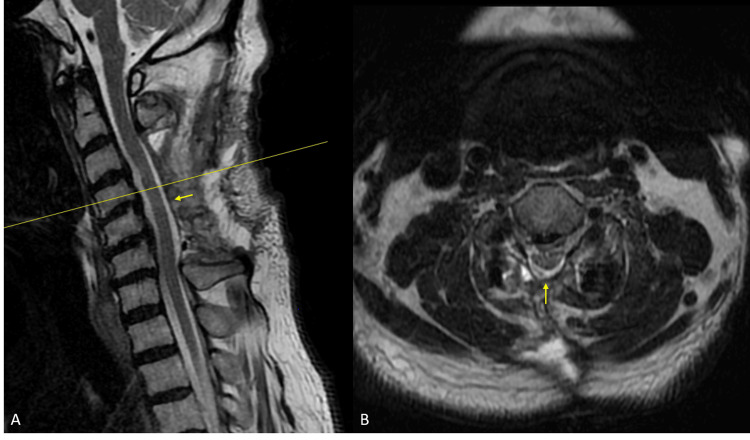
Postoperative sagittal (A) and axial (B) MRI demonstrating the re-expansion of the cervical thecal sac (yellow arrows) after drainage of the seroma

An emergency decompression was performed in the operating theater, and intraoperative findings were that of a large collection of clear fluid beneath the fascial layer that was under pressure. No purulence or active bleeding was noted, and the visualized thecal sac was well-filled with no leakage of cerebrospinal fluid (CSF) after a Valsalva maneuver. All intraoperative cultures were negative. The wounds were closed in layers over two suction drains placed beneath the fascia. A postoperative MRI scan was performed to confirm the re-expansion of the thecal sac (Figure 3, Panels A and B).

The patient’s neurological status gradually improved back to its baseline preoperative state, and at this time, the drains were only removed after drain output was minimal on postoperative days five and eight.

Case 2

**Figure 4:**
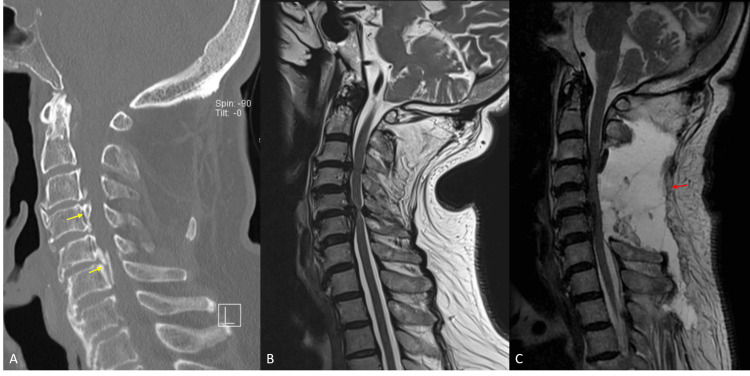
(A) Preoperative CT showing segmental OPLL (yellow arrows). (B) Preoperative MRI showing multilevel cord compression. (C) MRI on postoperative day six demonstrating compressive seroma (red arrow). OPLL: Ossification of the posterior longitudinal ligament.

A 76-year-old gentleman with a history of diabetes, hypertension, and hyperlipidemia presented to the emergency department with bilateral lower limb weakness and numbness for around a year with progressive gait difficulties over a week. He had been independently ambulant in the community with a walking stick premorbidly but became homebound over the past week as a result of his progressive gait unsteadiness. Significant findings on physical examination included bilateral lower limb weakness (4/5) accompanied by an upgoing plantar reflex in the right lower limb as well as significant ataxia with an inability to stand without support. Motor power was full in the upper limbs. Imaging revealed multilevel degenerative changes with segmental ossification of the posterior longitudinal ligament (OPLL) and severe cord compression over C3-C6 with cord signal changes at C5-C6 (Figure 4, Panels A and B).

He underwent C3-C7 laminectomy with C2-T1 instrumented fusion with the aim of preventing future progression of the OPLL mass. Human gelatine-thrombin matrix was used as a hemostatic agent in this case; otherwise, the surgical technique including graft choice and closure technique was followed as described in Case 1. There was a drop in right C5-C7 motor evoked potentials (MEP) following laminectomy, while the left upper limb MEP, bilateral lower limb MEP, and bilateral somatosensory evoked potentials (SSEP) all remained stable.

The patient woke up from surgery with no new neurological deficits. The drain was removed on day four after the drain output decreased to 30 mL over 24 hours. On postoperative day six, the patient was noted to have new-onset decreased motor power of his left shoulder abduction, elbow flexion and extension, and wrist extension (3/5). An urgent MRI was performed, which revealed a large T2 hyperintense fluid collection that was compressing the cord (Figure 4, Panel C).

An emergency decompression was performed in the operating theater, and intraoperative findings were similar to those of Case 1, with a large collection of clear fluid under pressure beneath the fascial layer. Again, no purulence, active bleeding, or leakage of CSF was noted, and all intraoperative cultures were negative. The wounds were closed in layers over two suction drains placed beneath the fascia.

The drains were removed on postoperative days five and six. The patient’s left upper limb weakness improved partially (4/5) following decompression of the seroma and subsequently recovered back to his preoperative baseline one month later. His ambulatory status improved following a period of rehabilitation. By four months postoperatively, he was able to regain independent ambulatory status.

Case 3

**Figure 5:**
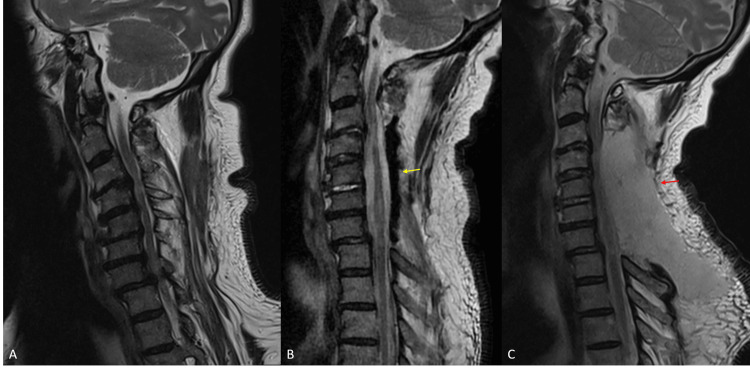
(A) Preoperative MRI demonstrating multilevel cord compression with mild kyphosis. (B) Immediate postoperative MRI demonstrating adequate posterior decompression (yellow arrow) and improved alignment. (C) MRI on postoperative day 11 showing a large compressive seroma (red arrow).

A 73-year-old gentleman with a known history of hypertension, hyperlipidemia, and cervical myelopathy, for which he had previously declined surgical intervention, presented to the emergency department with an incomplete cervical spinal cord injury (American Spinal Injury Association Impairment Scale grade C) following a fall onto his face on the same day. On admission, he was noted to have significant weakness in his bilateral upper and lower limbs (2-3/5) with a neurological level of injury at C5. Urgent MRI revealed multilevel degenerative changes with severe cord compression over C3-T1, cord signal changes at C3-C4 and C6-C7, and mild cervical kyphosis at C3-C5 (Figure 5, Panel A).

He underwent C3-T1 laminectomy and instrumented fusion. The surgical technique was identical to that described in Case 2. MEP and SSEP remained stable during surgery, with mild improvement in the left C6 MEP following decompression. A repeat MRI was obtained immediately postoperatively, which showed good decompression of the spinal cord and improved overall alignment of the cervical spine (Figure 5, Panel B). Postoperative progress was uneventful, and drains were removed on day five postoperatively when drain output had turned serous and decreased to 140 mL over 24 hrs. On postoperative day eight, the patient complained of neck pain. It progressed to severe neck and bilateral upper limb allodynia by postoperative day 11, which was incompletely relieved by opioid analgesics. His motor power remained similar to his preoperative baseline. In view of the increasing pain, an urgent MRI was performed, which revealed a large homogeneous T2 hyperintense fluid collection in the surgical site compressing the spinal cord (Figure 5, Panel C).

The patient was brought back into the operating theater for drainage of this fluid collection, and intraoperative findings were identical to those in Cases 1 and 2. Intraoperative cultures returned negative results. Surgical drains were subsequently removed on postoperative day seven. The patient’s pain improved by the second day following drainage of the seroma, and the rest of his subsequent rehabilitation was otherwise uneventful.

Case 4

**Figure 6:**
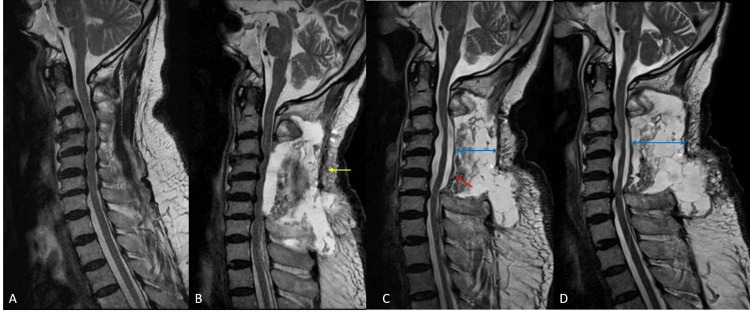
(A) Preoperative MRI demonstrating multilevel cord compression and mild kyphosis. (B) MRI on postoperative day seven demonstrating compressive seroma (yellow arrow). (C) Immediate post-aspiration MRI demonstrating re-expansion of thecal sac (red arrow) following seroma drainage. (D) MRI on post-aspiration day 10 showing a slight increase in the size of the seroma (blue arrows) without significant mass effect on the spinal cord.

A 75-year-old gentleman with a history of old stroke disease on aspirin, hypertension, and hyperlipidemia presented with gait unsteadiness and bilateral lower limb weakness of two weeks’ duration requiring the use of a walking stick for ambulation. Significant findings on physical examination included significant ataxia with an inability to stand without support as well as mild bilateral lower limb weakness (4/5). Imaging revealed a cervical kyphosis, which was correctable to <10 degrees on neck extension, as well as multilevel degenerative changes with severe cord compression over C3-C6 and myelomalacia at C3-C4 (Figure 6, Panel A).

He underwent C3-C6 laminectomy with C2-C6 instrumented fusion using lateral mass screws to allow correction of his cervical kyphosis to neutral alignment. The surgical technique was identical to that described in Case 1, and neuromonitoring was stable throughout.

The patient woke up from surgery with a stable neurological status compared to the preoperative condition. The drain was removed on postoperative day three after the drain output turned serous and drainage was 80 mL over 24 hrs. Initial rehabilitation was uneventful until the patient complained of mild aching pain in his right shoulder on postoperative day seven. He was noted to have slightly decreased motor power of bilateral elbow flexion and extension (3/5) and grip strength (4/5) compared to his examination on postoperative day one, and an urgent MRI was performed. This revealed a large T2 hyperintense fluid collection that was compressing the cord (Figure 6, Panel B).

Unlike in the preceding cases, an image-guided aspiration of the collection was performed, which yielded 75 mL of dark hemoserous fluid. No drain was placed. A post-aspiration MRI confirmed adequate drainage of the seroma (Figure 6, Panel C). The patient’s symptoms improved immediately after aspiration, and his motor power returned to his baseline immediately after the operation. He was able to recommence rehabilitation and achieved ambulation with a walking frame. However, over the next 10 days, the patient was noted to have fluctuating motor power in his upper limbs (3-4/5). A repeat MRI scan was performed, which showed re-accumulation of the fluid collection, although it was less sizable this time and there was less mass effect on the thecal sac (Figure 6, Panel D). A repeat image-guided aspiration was performed, which yielded 40 mL of hemoserous fluid, and a closed suction drain was inserted. The patient’s neurological status was maintained at baseline, and he was able to regain ambulatory status with a walking frame. The drain was removed on post-aspiration day nine. Fluid cultures from both aspirations were negative.

A summary of the four cases is presented in Table 1.

**Table 1:** Summary of cases DBM: Demineralized bone matrix; ORC: Oxidized regenerated cellulose; Vanco: Vancomycin; ASIA C: American Spinal Injury Association grade C.

Case	Age/Gender	Pre-op symptoms	Surgery	Intra-op adjuncts	Drain removal (days post-op)	Timing of deterioration (days post-op)	Post-op symptoms	Treatment
1	67/M	Left upper and lower limb dysfunction	C3-C6 laminectomy and fusion	DBM, ORC, Vanco powder	3	7	Bilateral upper limb weakness	Open drainage
2	76/M	Bilateral lower limb weakness and ataxia	C3-C7 laminectomy, C2-T1 fusion	DBM, gelatine-thrombin, Vanco powder	4	6	Left upper limb weakness	Open drainage
3	73/M	ASIA C spinal cord injury	C3-T1 laminectomy and fusion	Gelatine-thrombin, Vanco powder	5	8	Severe neck and bilateral upper limb pain	Open drainage
4	75/M	Bilateral lower limb weakness and ataxia	C3-C6 laminectomy, C2-C6 fusion	DBM, ORC, Vanco powder	3	7, 17	Bilateral upper limb weakness	Aspiration x 2

## Discussion

Seroma formation is a known complication of spinal surgery, which in the literature has been most commonly attributed to the use of rhBMP-2 [7,8]. While the incidence of asymptomatic seromas in the postsurgical field is likely to be under-reported due to postoperative MRI scans not routinely being performed in most cases, the formation of compressive seromas causing neurological compromise in the cervical spine is a much rarer entity, with only eight cases reported in the literature to our knowledge [1-6]. The focus on the use of rhBMP-2 in most reports perhaps underplays the risk of this complication in the absence of rhBMP-2.

Our four cases presented, which occurred over the course of a short two-year period, highlight that this complication may not be as rare as portrayed in the literature and does not solely occur with the use of rhBMP-2. The clinical course is typically one gradual and delayed neurological deterioration that begins several days postoperatively and after the removal of surgical drains, which would have prevented further fluid build-up, given the serous nature of the fluid. Other cases reported in the literature have described neurological deterioration occurring as early as three days and up to six weeks postoperatively [1-6,9]. Neurological deterioration in such cases is seldom dramatic and typically follows a more insidious course, making it easy for clinicians to attribute the symptoms to postoperative pain or generalized deconditioning, which may be common in the postoperative state, especially in elderly patients. A high index of suspicion and a low threshold for performing a postoperative MRI scan, even in the absence of severe symptoms or dense neurological deficits, likely contributed to the detection of this complication in the cases presented.

This typical sequence of events is in keeping with the pathophysiology of seroma formation, which authors have attributed to an inflammatory cytokine-mediated response resulting in soft tissue swelling and the production of fluid in the surgical field [2,7,8]. This fluid accumulates in the dead space created by surgery, and the formation of a fibrous capsule comprising granulation tissue around the fluid collection is believed to further impair its reabsorption postoperatively [10,11]. As fluid builds up in the surgical bed following drain removal and pressure on the spinal cord gradually increases, varying degrees of pain, weakness, and/or proprioceptive difficulties may result and manifest only several days to weeks after the index surgery.

The majority of papers on this subject have proposed rhBMP-2 as the main risk factor responsible for seroma formation. Instead, we postulate that in addition to rhBMP-2 use, a variety of other contributing factors may also increase the risk of compressive seroma formation. First, the use of instrumentation using an open technique, in which a wide subperiosteal elevation of the paraspinal muscles is performed, leaves a large dead space for seromas to accumulate. This has been previously raised by other authors as a likely risk factor [10], and we fully concur with this belief. In our experience, we have never encountered this complication in patients who have undergone open-door laminoplasty or non-instrumented laminectomies, in which there is much less dead space for seroma formation. Similarly, in the literature, there has only been a single case report of this complication that occurred following a laminectomy without instrumentation [6].

Second, the use of not only rhBMP-2 but, in fact, any biologically active foreign material, such as allograft [5], hemostatic agents [12], and/or vancomycin powder [9], in the surgical field have all been reported to increase the risk of seroma formation. In all these reports, the exact pathophysiology of seroma formation is unclear, although in most cases, authors have proposed that an inflammatory response elicited by these agents may be responsible. Some combination of the above agents was used in all of our cases, with only vancomycin powder being used in all four cases. We recommend weighing the benefits of each of these adjuncts carefully against their potential risks, with seroma formation being a specific consideration.

Finally, the fact that in all four cases, neurological deterioration only occurred after postoperative day five suggests that fluid accumulation in the surgical field likely only peaks beyond this time frame. Premature removal of surgical drains prior to this peak may increase the risk of compressive seroma formation. We recommend keeping surgical drains until daily fluid output is on a consistent downward trend and below 30 mL over 24 hours. In some cases, this may necessitate drains being kept longer than routinely performed.

Management of this complication lies first and foremost in its early detection, which begins with being aware of the typical sequence of events and having a high index of suspicion in a patient who may only have mild or non-specific but progressive symptoms in the postoperative course. Early drainage of the compressive seroma generally results in immediate symptomatic improvement. While we elected to re-open the wound for exploration and drainage in Cases 1 to 3, an image-guided percutaneous aspiration was performed in case 4, which yielded equally satisfying results without the morbidity of repeat surgery. This technique was described in a recent study [13], and we believe that such an approach may be beneficial in cases where the diagnosis of seroma is clear from postoperative imaging, but there is a low index of suspicion of other differential diagnoses such as an epidural hematoma or an occult CSF leak, which may necessitate formal re-opening of the wound.

We acknowledge the limitations of our study. Having only small case numbers without a control group, we are unable to draw any definitive conclusions regarding the causation of this complication. However, we believe that the similarities in the cases presented brought forth several hypotheses regarding the risk factors potentially predisposing to postoperative seromas as discussed earlier, which can then be investigated further in future larger-scale studies.

## Conclusions

These four cases add to the currently limited body of literature describing this complication and highlight the typical presentation of compressive postoperative seromas in the cervical spine. Our cases refute the prevailing understanding that rhBMP-2 is the main culprit for postoperative seroma formation, and we postulate that compressive seroma formation may not be as rare a complication as the literature seems to suggest, with multiple contributing risk factors. We urge clinicians to have a high index of suspicion in the event of gradual neurological deterioration in the days following drain removal, and advocate early MRI scan to confirm the diagnosis. Early drainage of the fluid collection allows good relief of compressive symptoms, and radiologically-guided aspiration may be a feasible alternative to open drainage in selected cases.
